# Evaluating burnout of soccer players with respect to their positions

**DOI:** 10.3389/fpsyg.2026.1781981

**Published:** 2026-04-13

**Authors:** Mine Taskin, Halil Taskin

**Affiliations:** 1 Ali Akkanat School of Applied Sciences, Selçuk University, Konya, Türkiye; 2 Sport Science Faculty, Selçuk University, Konya, Türkiye

**Keywords:** amateur, burnout, footballer, playing position, soccer

## Abstract

**Background:**

Athlete burnout is a common negative psychological state of athletes, which includes physical/emotional exhaustion, diminished personal accomplishment, and negative evaluation of sports. The aim of this study is to examine the burnout dimensions in amateur football players according to playing positions.

**Methods:**

A total of 113 amateur soccer players participated in the study (age range: 20–35) and the mean (SD) weight was 74.81 ± 8.41 kg, height was 179 ± 2.56 cm. An Athlete Burnout Questionnaire was used to determine the athlete burnout dimensions of soccer players.

**Results:**

The results of study show that the data revealed the defense players had higher values in the two subscales: emotional/physical exhaustion (EPE) and sport devaluation (SDeval). On the other hand, the strikers had higher values in the reduced sense of accomplishment (RSA) subscale.

**Conclusion:**

Based on the indicators of burnout dimensions, goalkeepers, midfielders, and forwards appear to make adequate psychophysiological adjustments to the demands of competition regarding physical and emotional exhaustion and sport devaluation. In terms of reduced sense of accomplishment, goalkeepers, defenders, and midfielders demonstrate sufficient psychophysiological adaptation to competitive demands. The impact (preventing goals and scoring goals) represented by defenders and strikers with higher burnout scores should be investigated.

## 1 Introduction

The concept of burnout was first scientifically defined in the 1970s (Freudenberger, 1974; Maslach, 1976) and has since become a recognized phenomenon in many cultures and settings, including the athletic community. Freudenberger (1974), described burnout as a state of physical and emotional exhaustion accompanied by a sense of failure. The most frequent reasons are high levels of stress, overwork, lack of control, poor work relationships, and lack of support in the work environment (Freudenberger, 1974). Burnout is defined as a syndrome of emotional exhaustion and cynicism that is frequently seen in individuals working in the field of human services. When people feel burned out, they believe they have exhausted their psychological resources and develop cynical and negative attitudes toward others and a negative view of themselves (Maslach and Jackson, 1981). Raedeke (1997), who first introduced the concept of athlete burnout in competitive sports, defined it as a syndrome characterized by a decreased sense of accomplishment, emotional/physical fatigue, and negative self-evaluation. Burnout in sports refers to a response to chronic stress in athletes, characterized by a decline in performance and physical and emotional exhaustion (Cresswell and Eklund, 2006). Some athletes quit sports due to burnout, a syndrome characterized by a diminished sense of accomplishment and a devaluation of sport participation. Decreased motivation and the cessation of sport participation are also characteristics of burnout (Verardi et al., 2014). While burnout in sports refers to a response to chronic stress in athletes, burnout is more than a simple stress response; among competitive athletes, the number of athletes experiencing high levels of burnout is estimated to be between 1% and 9%, while the number of athletes suffering from severe burnout is estimated to be 1%−2% (Gustafsson, 2007). This demonstrates that not all athletes experiencing stress burn out (Gustafsson, 2007). Athlete burnout is a common negative psychological state of athletes, which includes physical/emotional exhaustion, diminished personal accomplishment, and negative evaluation of sports. The pressure created by elite soccer players' daily desire to perform at the highest level and the obligation to be a role model puts the modern soccer player at risk of burnout and it was the last stage of psychological collapse when athletes suffered from adverse pressure for a long time (Smith et al., 2020). In soccer players, professional burnout consists of a decline in athletic skills and performance, physical and emotional exhaustion from training and competition, loss of self-confidence, and a lack of value and interest (Kimberley et al., 2011; Lemyre et al., 2008). Soccer involves different technical and tactical characteristics in terms of playing positions. Defenders position themselves in relation to the ball, their teammates, and the opponent to defend the goal and disrupt the opponent's possession (Guimarães et al., 2014). Midfielders, on the other hand, establish the link between defenders and forwards, making transitions from defense to attack and from attack to defense. Forwards, unlike defenders, have attacking characteristics that help their team win (Gonçalves et al., 2017). Considering that different playing positions in football have different psychological characteristics, they may have different sensitivities to the sub-dimensions of burnout perception (Junge et al., 2000). We found that most of the existing studies focus on the relationship between factors, but there were few studies on playing positions of amateur soccer players. Unlike professional soccer players, amateur soccer players often juggle other important life responsibilities alongside their sport, thus balancing work/school with sport or training. This dual-career structure may create additional time constraints and stressors, which may cause higher levels of burnout, including emotional exhaustion and reduced sense of accomplishment, among amateur soccer players. Therefore, the aim of the present study was to analyze the components of burnout (physical and emotional exhaustion, sport devaluation and decreased sense of accomplishment) in amateur soccer players' playing positions. With generally, burnout levels differ significantly according to playing positions among amateur soccer players. Sub-hypotheses: (a) Physical and emotional exhaustion scores differ significantly according to playing positions among amateur soccer players, (b) Sport devaluation scores differ significantly according to playing positions among amateur soccer players, and (c) Decreased sense of accomplishment scores differ significantly according to playing positions among amateur soccer players.

## 2 Methods

### 2.1 Subjects

A total of 113 amateur male soccer players aged 20–35 years participated in the study. The mean (SD) body weight was 74.81 ± 8.41 kg, and the mean (SD) height was 179 ± 2.56 cm. Participants were competing in the first amateur league, super amateur league, and regional amateur league. The sample consisted of 21 goalkeepers (first amateur league, *n* = 7; super amateur league, *n* = 7; regional amateur league, *n* = 7), 27 defenders (first amateur league, *n* = 9; super amateur league, *n* = 9; regional amateur league, *n* = 9), 32 midfielders (first amateur league, *n* = 15; super amateur league, *n* = 6; regional amateur league, *n* = 11), and 33 strikers (first amateur league, *n* = 14; super amateur league, *n* = 11; regional amateur league, *n* = 8). Soccer players had a mean playing experience of 12.07 ± 3.27 years. Data collection was conducted during the competitive season, during which all participants were actively competing, and they reported an average weekly training load of 6 h per week, reflecting their regular in-season training routines.

### 2.2 Procedure

The study employed a “Survey Methodology,” and data were collected through an online survey. An Athlete Burnout Questionnaire was used to determine the athlete burnout scores of soccer players. The online survey link was sent to club coaches via WhatsApp. The coaches then sent the link to the players via WhatsApp, allowing them access to the survey. The completed online surveys were automatically saved to Google Drive. The collected survey data were organized by the authors and prepared for statistical analysis.

#### 2.2.1 Athlete burnout questionnaire

It was developed by Raedeke and Smith (2001) to determine the burnout levels of athletes. The original scale consists of 3 subscales, each containing 5 items (emotional/physical exhaustion (items: 1-3-7-9-10), sport devaluation (items: 2-5-8-13), and decreased sense of accomplishment (items: 4-6-11-12). The Turkish adaptation of the Athlete Burnout Scale, which is assessed using a 5-point Likert type, was conducted by Kelecek et al. (2017). Due to low factor loadings in the adaptation study, 2 items were removed from the scale. These items are item 1 (I achieve many valuable things in sports) from the decreased sense of accomplishment sub-dimension and item 11 (I worry less about succeeding in sports than before) from the sport devaluation sub-dimension. The final Turkish form of the Athlete Burnout Scale was determined to have 13 items (4 items in the decreased sense of accomplishment and sport devaluation subscales, and 5 items in the emotional/physical exhaustion subscale). In the Turkish adaptation study of the scale, Cronbach's Alpha internal consistency coefficients were reported as 0.87 for the emotional/physical exhaustion subscale, 0.75 for the decreased sense of accomplishment subscale, and 0.83 for the sport devaluation subscale (Kelecek et al., 2017).

#### 2.2.2 Inclusion and exclusion criteria

Amateur status was players who perform sporting activities without expecting financial or similar compensation.

Inclusion criteria were male amateur soccer players aged 18–35 years, actively competing in the first amateur, super amateur, or regional amateur leagues during the competitive season. In this study, soccer players were included based on their playing positions, while league-level classification was not considered as an inclusion criterion. Accordingly, soccer players were not categorized or analyzed according to the leagues.

Exclusion criteria were incomplete or inconsistent questionnaire responses, duplicate questionnaire responses, participation outside the competitive season, soccer players have injuries that prevent them from actively participating in competitions, and professional player status.

### 2.3 Statistical analysis

The IBM SPSS 22 software package was used to analyse the data obtained. Data were summarized as mean and standard deviation. The suitability of the data for normal distribution was examined with the Kolmogorov–Smirnov test. The test results for EPE, SDeval, RSA, and overall burnout were found as (*D* = 1.292, *P* > 0.05; *D* = 1.296, *P* > 0.05; *D* = 1.198, *P* > 0.05; *D* = 1.131, *P* > 0.05), respectively (Table 1), and the data were determined to be normally distributed. One-Way ANOVA was used to compare playing positions of amateur soccer players related to value for burnout in the three sub-scales of the Athlete Burnout Questionnaire. The Tukey multiple comparison test was used to test which position caused the difference, based on homogeneity of variance (equal variances). According to the Levene test results, *F*_(3, 109)_ = 1.894, *P* > 0.05 for EPE; *F*_(3, 109)_ = 2.024, *P* > 0.05 for SDeval; *F*_(3, 109)_ = 2.424, *P* > 0.05 for RSA; and *F*_(3, 109)_ = 1.696, *P* > 0.05 for overall burnout (Table 1). The error level in this study was set at 0.05.

**Table 1:** Levene's test for homogeneity of variance and Kolmogorov–Smirnov test statistics.

Variables to cell combination structures		Levene test					Kolmogorov–Smirnov test	
		Levene statistic		Df1	Df2	*P*	*D*	*P*
Burnout sub-dimensions (score)	EPE	Based on Mean	1.894	3	109	0.135	1.292	0.075
	SDeval	Based on Mean	2.024	3	109	0.115	1.296	0.071
	RSA	Based on Mean	2.424	3	109	0.70	1.298	0.069
Overall burnout		Based on Mean	1.696	3	109	0.173	1.294	0.073

The test results for EPE, SDeval, RSA, and overall burnout were found as (D = 1.292, P > 0.05; D = 1.296, P > 0.05; D = 1.298, P > 0.05; D = 1.294, P > 0.05), respectively, and the data were determined to be normally distributed. According to the Levene test results, F_(3, 109)_ = 1.894, P > 0.05 for EPE; F_(3, 109)_ = 2.024, P > 0.05 for SDeval; F_(3, 109)_ = 2.424, P > 0.05 for RSA; and F_(3, 109)_ = 1.696, P > 0.05 for overall burnout.

## 3 Results

The suitability of the data for normal distribution was examined with the Kolmogorov–Smirnov test.

Table 2 shows the mean and standard deviations of the soccer players' burnout scores by playing positions for 113 soccer players. When burnout scores were examined in terms of playing position, defensive players had the highest scores in EPE, SDeval, and overall burnout, while forwards had the highest scores in RSA. On the other hand, midfielders were found to have the lowest scores in SDeval, RSA, and overall burnout, while goalkeepers had the lowest score in EPE.

**Table 2:** The mean and standard deviations of the soccer players' burnout scores by playing positions.

Variables	Burnout sub-dimensions (score)			
	EPE	SDeval	RSA	Overall burnout
Playing positions	Mean ±SD	Mean ±SD	Mean ±SD	Mean ±SD
Goalkeeper (*n* = 21)	8.05 ± 2.78	7.14 ± 2.71	10.48 ± 2.64	25.66 ± 5.55
Defense (*n* = 27)	11.85 ± 4.57	9.04 ± 3.39	9.70 ± 1.81	30.59 ± 8.50
Midfielder (*n* = 32)	9.25 ± 3.52	6.38 ± 2.64	9.47 ± 2.37	25.09 ± 6.95
Striker (*n* = 33)	10.00 ± 4.11	8.24 ± 3.95	11.21 ± 2.69	29.45 ± 9.53

Significant playing positions differences were found for EPE (η^2^*p* = 0.106), SDeval (η^2^*p* = 0.108), RSA (η^2^*p* = 0.079), and overall burnout (η^2^*p* = 0.088), indicating moderate to large effect sizes. *Post hoc* analyses revealed that defenders scored significantly higher than goalkeepers in EPE (*P* < 0.05, *d* = 3.80), and higher than midfielders in SDeval (*P* < 0.05, *d* = 2.94) and overall burnout (*p* < 0.05, *d* = 7.67). Additionally, strikers scored higher than midfielders in RSA (*p* < 0.05, *d* = 2.71). Overall, the results show significant differences based on playing positions, with defenders consistently scoring higher than other playing positions and midfielders generally showing lower scores (Table 3).

**Table 3:** Comparison of burnout scores of soccer players according to playing position (ANOVA).

Variables	Playing positions	Sum of squares	*df*	Mean square	*F*	*P*
EPE	Between groups	184.708	3	61.569	4.294	0.007^*^
	Within groups	1,563.026	109	14.340		
SDeval	Between groups	131.626	3	43.875	4.381	0.006^*^
	Within groups	1,091.595	109	10.015		
RSA	Between groups	59.620	3	19.873	3.210	0.026^*^
	Within groups	674.928	109	6.192		
Overall burnout	Between groups	641.682	3	213.894	3.509	0.018^*^
	Within groups	6,644.336	109	60.957		

^*^p < 0.05.

**Figure 1:**
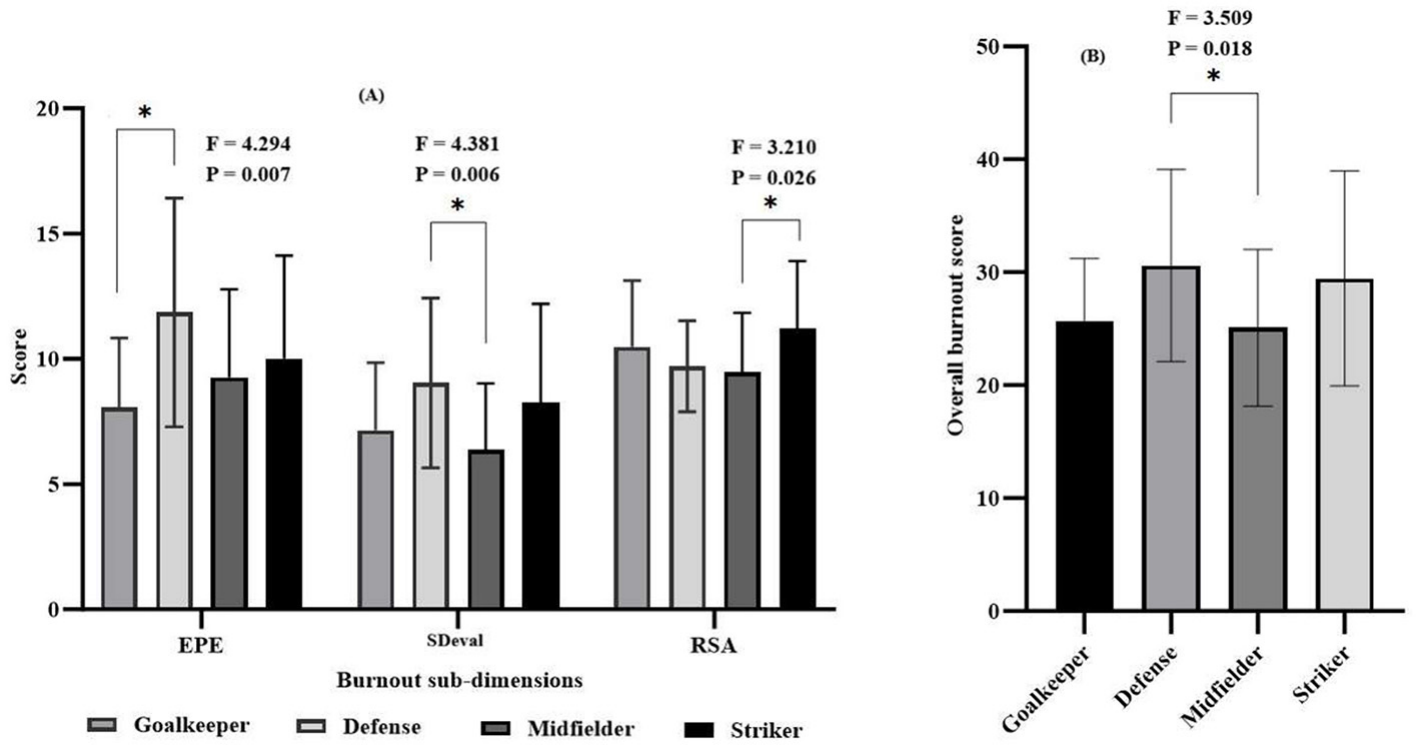
Burnout sub-dimension **(A)** and overall burnout **(B)** scores of soccer players with respect to their play positions. *p < 0.05.

When Figure 1 is examined, the data revealed the defense players had higher values in the two subscales: EPE (11.85); SDeval (9.04), On the other hand, the strikers had higher values in the RSA (11.21) subscale. Error bars in the figure were as SD; 95% CI.

## 4 Discussion

This study, aimed to examine burnout variables in amateur soccer players by position, found that defenders had higher EPE and SDeval burnout scores. The higher EPE and SDeval of defenders is thought to stem from the pressure of defensive responsibilities and preventing goals. The fact that defensive contribution is mostly defined through preventing mistakes, and that this contribution is relatively low in visibility, while mistakes receive a high degree of attention, can pave the way for the development of cynicism in players over time. It was also found that forwards had higher RSA burnout scores. This suggests that they may face greater pressure to meet visible performance metrics, particularly the expectation to score goals. In terms of total burnout, defenders were found to have higher burnout. The results of this study may be interpreted within established theoretical frameworks such as Smith's Cognitive-Affective Stress Model and Self-Determination Theory, which emphasize the role of cognitive appraisal, emotional responses, and basic psychological needs in shaping behavioral and performance-related outcomes. De Francisco et al. (2025) reported that the implementation of interventions aimed at enhancing self-determined motivation and satisfying athletes' basic psychological needs is essential for reducing the risk of burnout in sports contexts.

Previous research has shown that when athletes experience burnout, they suffer from a lack of motivation, their interest in training decreases, they become extremely tired from training, and may even exhibit symptoms of anxiety and depression, which negatively impacts their mental health (Demirci and Çepikkurt, 2018). Bicalho et al. (2020) reported that the highest number of athletes with elevated burnout levels (*n* = 17) was observed during the training period. Pires et al. (2019) and Hill et al. (2008) examined burnout in soccer athletes, generally reporting moderate to low levels across dimensions. Nevertheless, a small proportion of athletes exhibited more pronounced symptoms, particularly in reduced sense of sports achievement and physical and emotional exhaustion. Burnout-related variables during the preparatory period among professional and amateur players have been examined across playing positions, revealing that professional goalkeepers and forwards, as well as amateur defenders, exhibited the highest mean total burnout scores, particularly in relation to physical and emotional exhaustion. However, when professional and amateur players were compared within the same positional roles, no significant differences were observed across the three dimensions of burnout (Verardi et al., 2014). These findings suggest that positional demands, rather than competitive level alone, may play a more critical role in shaping burnout experiences among athletes. Values associated with greater vulnerability and, consequently, greater risk of evolving to the syndrome, were observed in a small portion of the players interviewed (Verardi et al., 2015). The results of a previous study show that many athletes had low burnout indicators, there was no difference in the perception of burnout dimensions by playing position, and the prevalence of athletes with burnout, whether mild, moderate, or severe, was 13% (Ferreira et al., 2021). Granz et al. (2019) suggested that athletes with low levels of subjective stress outside of sport and effective coping strategies tend to report lower scores on the physical and emotional exhaustion dimension. Professional goalkeepers also have higher SDeval scores compared with amateur goalkeepers and all other playing positions. Goalkeepers' special qualities and psychological preparation as well as their greater experience may make them more susceptible to physical and psychological pressure compared with the other positions (Brandão, 2000; Mahl and Raposo, 2007). Mindfulness and emotion regulation strategies have also been shown to mitigate the impact of life stress on burnout, particularly in adolescent athletes, emphasizing the importance of developing cognitive-affective skills alongside physical training (Ma et al., 2025). Accordingly, mindfulness and emotion regulation interventions could be tailored to the specific demands of playing positions, with a greater focus on managing performance anxiety and outcome-related pressure in strikers and addressing sustained cognitive load and chronic performance pressure in defenders. A meta-analysis exam currently implemented psychological interventions to reduce or eliminate burnout syndrome in young athletes reviewed five studies. This meta-analysis found that most dimensions of burnout were effectively reduced with cognitive-behavioral therapy and mindfulness-based interventions. Furthermore, online interventions were found to play a significantly greater role in this reduction (Wilczyńska et al., 2022).

## 5 Conclusions

In conclusion, the results of our study revealed that defensive players had higher values in two subscales: EPE (11.85) and SDeval (9.04). On the other hand, the strikers had higher values in the RSA (11.21) subscale. Based on the indicators of burnout dimensions, goalkeepers, midfielders, and forwards appear to make adequate psychophysiological adjustments to the demands of competition regarding physical and emotional exhaustion and sport devaluation. In terms of reduced sense of accomplishment, goalkeepers, defenders, and midfielders demonstrate sufficient psychophysiological adaptation to competitive demands. The impact (preventing goals and scoring goals) represented by defenders and strikers with higher burnout scores should be investigated.

## 6 Practical applications

Coaches working with players in high-risk positions should carefully monitor workload, promote autonomy, and provide stress management support. Sport psychologists can design position-specific interventions that address both cognitive-affective stress and motivation, while team management should implement systematic monitoring and early intervention protocols to safeguard player wellbeing. Together, these strategies help prevent burnout and maintain both performance and psychological health. Amateur coaches can implement practical tools such as Rating of Perceived Exertion (RPE) scales to monitor perceived training intensity, and brief wellness questionnaires to assess athletes' recovery status across domains such as fatigue, sleep quality, muscle soreness, and mood. These tools provide a low-cost and easily applicable method for tracking training load and identifying early signs of excessive stress without the need for specialized medical staff.

## 7 Limitations and future research directions

This study has a cross-sectional design, and the findings reflect the relationships observed at a single point in time. Therefore, the results should not be interpreted as causal relationships. However, the cross-sectional design offers significant value in revealing the current state of the variables under study and describing the differences between groups instantaneously. The findings can form a basis for future longitudinal or experimental studies and contribute to understanding the subject in its current context.

Firstly, this study chose Turkish amateur soccer players as research objects, which is not suitable for other age groups or other sports. Future research could be extended to other age groups.

Although previous research has shown some gender differences in burnout between male and female athletes, only male soccer players participated in this study. Therefore, it is recommended that subsequent researchers extend the study to examine changes in burnout or explore other sport branches to help fill in the research gaps.

## Data Availability

The raw data supporting the conclusions of this article will be made available by the authors, without undue reservation.
